# Short-Term Administration of Lemon Balm Extract Ameliorates Myocardial Ischemia/Reperfusion Injury: Focus on Oxidative Stress

**DOI:** 10.3390/ph15070840

**Published:** 2022-07-08

**Authors:** Nevena Draginic, Isidora Milosavljevic, Marijana Andjic, Jovana Jeremic, Marina Nikolic, Jasmina Sretenovic, Aleksandar Kocovic, Ivan Srejovic, Vladimir Zivkovic, Sergey Bolevich, Stefani Bolevich, Svetlana Curcic, Vladimir Jakovljevic

**Affiliations:** 1 Department of Pharmacy, Faculty of Medical Sciences, University of Kragujevac, 34000 Kragujevac, Serbia; nevenasdraginic@gmail.com (N.D.); andjicmarijana10@gmail.com (M.A.); jovana.jeremic@medf.kg.ac.rs (J.J.); salekkg91@gmail.com (A.K.); 2 Department of Human Pathology, First Moscow State Medical University I.M. Sechenov, 119991 Moscow, Russia; bolevich2011@yandex.ru; 3 Department of Physiology, Faculty of Medical Sciences, University of Kragujevac, 34000 Kragujevac, Serbia; marina.rankovic.95@gmail.com (M.N.); drj.sretenovic@gmail.com (J.S.); ivan_srejovic@hotmail.com (I.S.); vladimirziv@gmail.com (V.Z.); 4 Department of Pharmacology of the Institute of Biodesign and Complex System Modelling, First Moscow State Medical University I.M. Sechenov, 119991 Moscow, Russia; 5 Department of Patophysiology, First Moscow State Medical University I.M. Sechenov, 119991 Moscow, Russia; alistra555@mail.ru; 6 Department of Pharmacology, First Moscow State Medical University I.M. Sechenov, 119991 Moscow, Russia; 7 Faculty of Education in Jagodina, University of Kragujevac, 34000 Kragujevac, Serbia; ceca.curcic64@gmail.com

**Keywords:** *Melissa officinalis* L., ethanolic extract, ischemia/reperfusion injury, rat heart, oxidative stress

## Abstract

We aimed to investigate the cardioprotective effects of ethanolic *Melissa officinalis* L. extract (ME) in the rat model of myocardial ischemia/reperfusion (I/R) injury. Thirty-two *Wistar* rats were randomly divided into a CTRL non-treated control group with myocardial I/R injury and three experimental groups of rats treated with 50, 100, or 200 mg/kg of ME for 7 days per os. Afterward, hearts were isolated, and cardiodynamic function was assessed via the Langendorff model of global 20 min ischemia and 30 min reperfusion. Oxidative stress parameters were determined spectrophotometrically from the samples of coronary venous effluent (O_2_^−^, H_2_O_2_, TBARS, and NO_2_^−^,) and heart tissue homogenate (TBARS, NO_2_^−^, SOD, and CAT). H/E and Picrosirius red staining were used to examine cardiac architecture and cardiac collagen content. ME improved cardiodynamic parameters and achieved to preserve cardiac architecture after I/R injury and to decrease fibrosis, especially in the ME200 group compared to CTRL. ME200 and ME100 markedly decreased prooxidants TBARS, O_2_^−^, and H_2_O_2_ while increasing NO_2_^−^. Hereby, we confirmed the ME`s ability to save the heart from I/R induced damage, even after short-term preconditioning in terms of preserving cardiodynamic alterations, cardiac architecture, fibrosis, and suppressing oxidative stress, especially in dose of 200 mg/kg.

## 1. Introduction

During the last few decades, myocardial ischemia has been recognized as the most common form of cardiovascular disease, causing still rising morbidity and mortality worldwide. Even though the restoration of blood flow is essential for survival and better patient outcome, substantial damage to the cardiomyocytes occurs during reperfusion, consequently inducing arrhythmias, myocardial stunning, microvascular dysfunction, inflammatory response, and ultimately cardiomyocyte death. This phenomenon is called myocardial ischemia/reperfusion (I/R) injury and has received extensive scientific interest for more than three decades globally [1]. The pathogenesis of myocardial I/R injury is very complex and includes mediation by a number of mechanisms, such as increased reactive oxygen species (ROS) generation leading to oxidative stress, mitochondrial dysfunction, intense inflammatory response, increased mitochondrial permeability transient pore (mPTP) opening, and calcium overload. All of these mechanisms induce cardiomyocyte` death either by necrosis, apoptosis, or autophagy [2]. Since therapeutic options for this state are limited to revascularization or fibrinolytic therapy, the role of natural products, especially plant extracts or isolated plant compounds, such as heart preconditioning agents, is gaining popularity in research society. The phytotherapeutical approach has drawn attention owing to its safety, lesser side effects, and cost-effectiveness [3]. Plants and plant derivatives` beneficial effects are mostly based on their strong antioxidant, free radical scavenging, and anti-inflammatory properties, which can be very useful in myocardial I/R injury pathology [4].

*Melissa officinalis* L. (*Lamiaceae*), commonly known as lemon balm or bee balm, is a perennial herb, which is a part of the mint fragrance family of plants. It is a long-known folk medicine commonly used as a sedative or calming agent for anxiety problems, memory enhancement, and heart palpitations [5]. Today it is known that MEs have rich phenolic content, including rosmarinic acid, chlorogenic acid, cinnamic acid, ferulic acid, and caffeic acid. Additionally, several flavonoids have been identified as the components of lemon balm extracts, such as luteolin, apigenine, rutin, quercetin, querctirin, catechine, and epicatechine, but also volatile compounds (monoterpens and sesquiterpens), and taninns to a lesser extent [5]. It is considered that the health-promoting effects of ME originate both from their phenolic content, especially rosmarinic acid as a major one, but also from flavonoid components. A vast number of studies extensively studied and confirmed the beneficial effects of certain flavonoids of ME in myocardial I/R injury, including quercetin [6,7,8], luteolin [9], apigenin [10,11], and catechine [12] via a number of mechanisms including antioxidant, antiapoptotic, anti-inflammatory, immuno-regulating effects. Additionally, phenolics, especially rosmarinic acid per se, which is considered a most important ingredient of ME, have meaningful I/R injury alleviating effects in different organs, not only heart [13,14] but also liver [15], lungs [16], and kidney [17]. Since ME represents a mixture of these valuable secondary metabolites, we speculated on its cardioprotective potential.

Preclinical investigations demonstrated beneficial effects of ME in the cardiovascular system using different animal models, including isoproterenol-induced infarction [18], left anterior descending artery ligation (LAD) model [19], and doxorubicin-induced cardiotoxicity [20]. The majority of these studies emphasized the ability of ME to decrease heart rate, slower heart conductivity, reduce the rate of ventricular arrhythmias post-infarction, and reduce the infarct size. One of the main proposed mechanisms of cardioprotection induced by ME refers to its strong antioxidant action, free radical scavenging ability, and amelioration of oxidative stress, but also anti-inflammatory and antiapoptotic action [21,22]. However, the data regarding cardioprotective effects of ME in I/R injury pathology are conflicting in terms of dosing and the duration of preconditioning [18,19].

Taking into account the abovementioned findings, this study aimed to provide new insight into the cardioprotective effects of ethanolic ME in the rat model of myocardial I/R injury by evaluating the cardiac function, morphology of the heart tissue, and the involvement of oxidative stress after short-term pre-treatment with ME in three different doses.

## 2. Results

### 2.1. Chemical Characterisation of the Examined Extract

All data regarding the chemical characterization of the investigated extract (ME), including HPLC (high-performance liquid chromatography) analyses, phenolic, and flavonoid content are provided in Supplementary Materials (IPublished) [23].

### 2.2. Effects of Heart Preconditioning with ME on Cardiac Function

**Figure 1:**
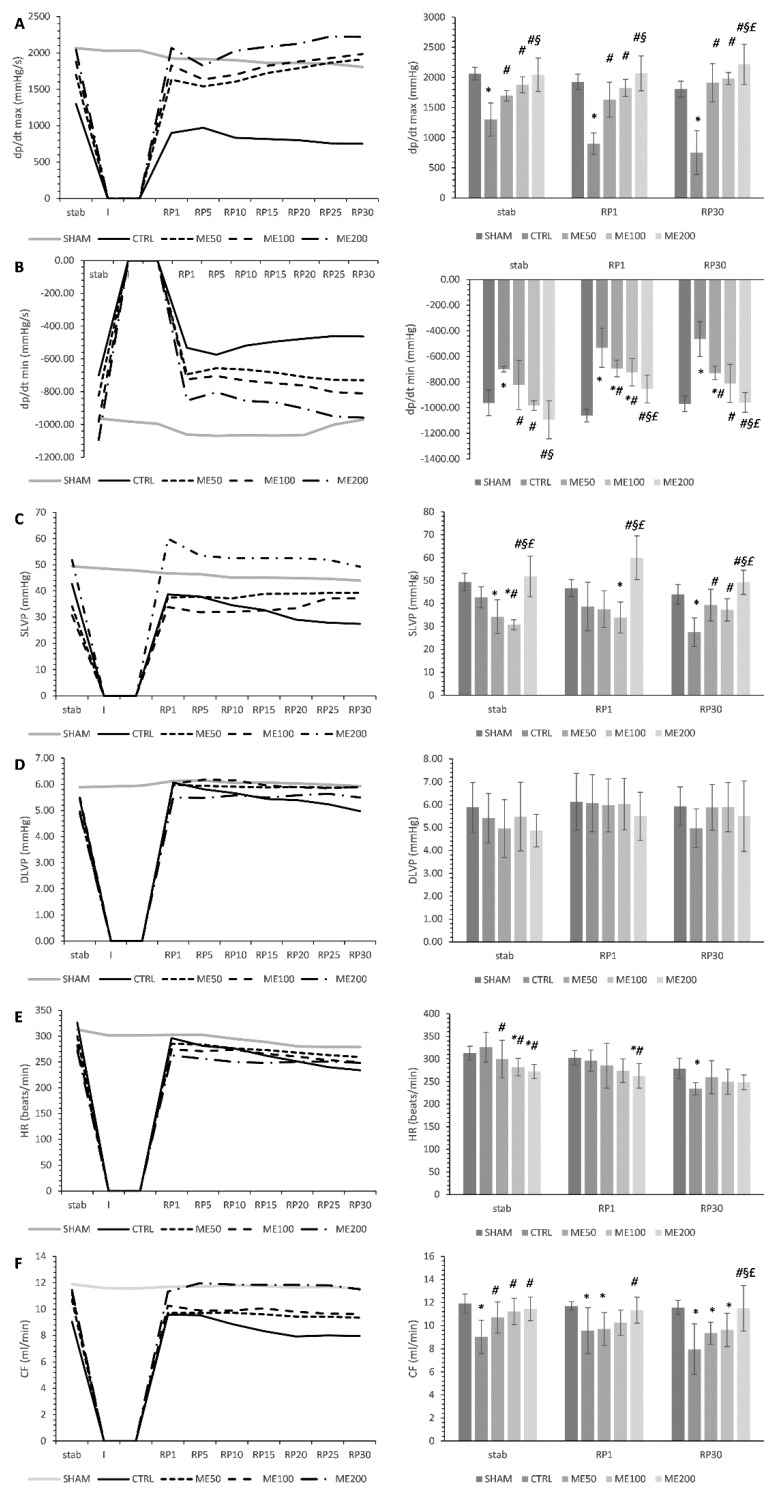
Effect of heart preconditioning with ME on the parameters of cardiac function. (**A**) Maximum rate of left ventricle pressure development−dp/dt max; (**B**) minimum rate of left ventricle pressure development−dp/dt min; (**C**) systolic left ventricle pressure−SLVP; (**D**) diastolic left ventricle pressure−DLVP; (**E**) heart rate−HR; (**F**) coronary flow−CF. All measured cardiodynamic parameters during whole ex vivo protocol are presented on the line graphs (on the left), while the points of interest (stab-stabilization; RP1−1st min of reperfusion; RP30−30th min of reperfusion) used for statistical analysis are presented on the bar graph (on the right). Values are expressed as means ± standard deviation (*n* = 8). *—statistical significance at the level of *p* < 0.05 compared to SHAM; #–statistical significance at the level of *p* < 0.05 compared to CTRL; §–statistical significance at the level of *p* < 0.05 compared to ME50 group; £—statistical significance at the level of *p* < 0.05 compared to ME100 group.

Parameters of contractile function minimum and maximum rate of left ventricle pressure development (dp/dt max and dp/dt min) were characteristically impaired after ischemia in the CTRL group compared to SHAM in all three points of interest stab, RP1, and RP30. Additionally, ME treatment affected the hearts’ ability to stabilize, as shown via significantly higher dp/dt max of all treated animals (ME50, ME100, and ME200) compared to CTRL and SHAM in stab point (*p* < 0.05). All three doses of ME succeeded in improving dp/dt max in all three points of interest compared to CTRL, while the most prominent effect was observed in the ME200 group, which showed significantly higher values of dp/dt max compared to CTRL, ME50, ME100 in both stabs, RP1, and RP30 points, with no significant difference when compared to SHAM (Figure 1A).

Similar changes were observed in the dp/dt min parameter, with all three experimental groups increasing this parameter values in stab, RP1, and RP30 points when compared to the CTRL group of rats. The highest dose of the applied extract ME200 induced the greatest increase in dp/dt min in all observed points of interest compared to CTRL, ME50, and ME100 groups. Additionally, only the ME200 group improved dp/dt min during the reperfusion period, similar to SHAM non-exposed to ischemia. Baseline values in stab point were affected by ME treatment, shown via increased values of dp/dt min compared to CTRL (Figure 1B, *p* < 0.05).

Baseline values of SLVP at stab point were significantly lower in ME50 and ME100 compared to SHAM, while ME200 stabilized the heart, similar to the healthy non-treated SHAM group. Ischemia caused a systolic dysfunction, as measured by systolic left ventricular pressure (SLVP) decrement throughout the reperfusion period in the CTRL group, especially at the end of reperfusion RP30 compared to the SHAM group, while in experimental groups, the trend was the opposite, SLVP remained more-less constant during the reperfusion period. Additionally, the ME200 group exerted the most prominent effect on SLVP, significantly increasing this parameter in all points of interest compared to both SHAM, CTRL, and lower doses of ME (ME50 and ME100) (Figure 1C, *p* < 0.05).

Diastolic left ventricular pressure (DLVP) was mildly lower in the CTRL group during the post-ischemia period in the RP1 and RP30 compared to the stab. However, the treatment with ME did not cause any significant changes in this parameter compared to the CTRL or SHAM in any of the observed points of interest. Additionally, in the experimental groups, DLVP showed a constant trend during the whole reperfusion period (Figure 1D).

Heart rate (HR) was significantly lowered in all three experimental groups treated with ME compared to the CTRL group prior to global heart ischemia in the stab point. Baseline values of only ME50 and ME100 were significantly lower in relation to the SHAM group in stab point. However, after 20 min ischemia, only in ME200 significantly lower HR remained constant compared to the CTRL, while at the end of reperfusion, these effects were lost. There was no significant difference in HR among all groups studied (Figure 1E, *p* <0.05).

At the beginning of the experimental protocol, in the stabilization period, all three experimental groups (ME50, ME100, and ME200) induced a significant elevation of coronary flow (CF) compared to the non-treated CTRL group (*p* < 0.05), with no differences between different doses of ME and no differences compared to SHAM. However, after a period of ischemia, in the 1st min of the reperfusion period, CF was significantly higher only in the ME200 group compared to CTRL (*p* < 0.05), while in the 30th min of reperfusion, this difference was even more visible, with ME200 showing significantly higher CF compared to CTRL, ME50, and ME100 groups. Only ME200 succeeded in improving CF, similar to values of the non-treated SHAM group of rats who were not exposed to ischemia in RP30 point (Figure 1F, *p* < 0.05).

### 2.3. Effects of Heart Preconditioning with ME on Cardiac Redox State

**Figure 2:**
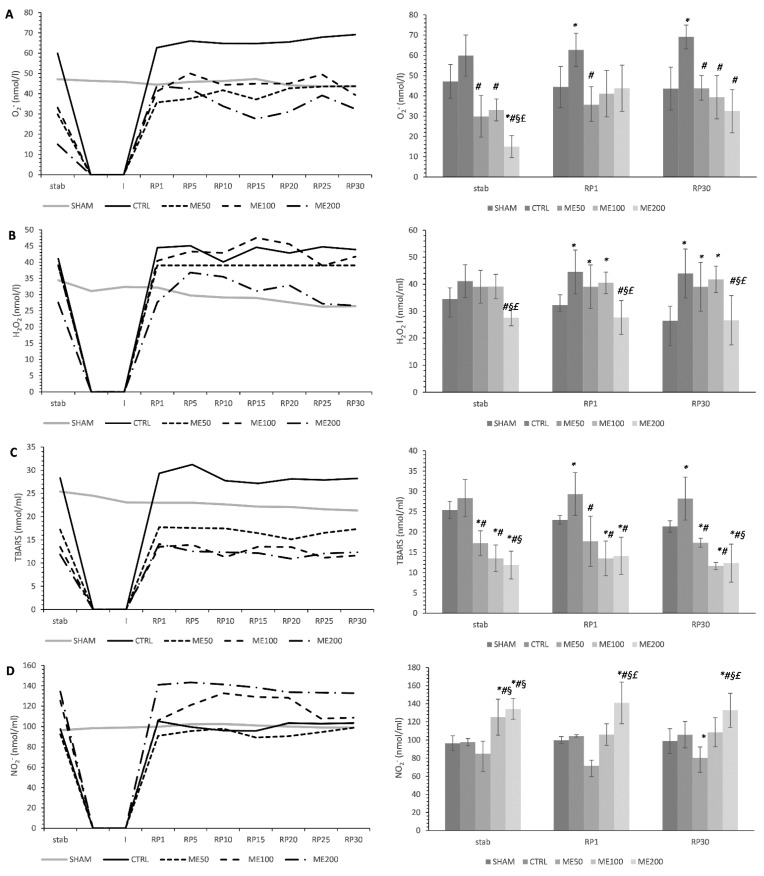
Effects of short-term heart preconditioning with ME on oxidative stress parameters in the coronary venous effluent. (**A**) O_2_^−^—superoxide anion radical; (**B**) H_2_O_2_—hydrogen peroxide; (**C**) TBARS—index of lipid peroxidation measured as thiobarbituric acid reactive substances; (**D**) NO_2_^−^—nitrites. All measured parameters of oxidative stress during whole ex vivo protocol are presented on the line graphs (on the left), while the points of interest (stab—stabilization; RP1—1st min of reperfusion; RP30—30th min of reperfusion) used for statistical analysis are presented on the bar graph (on the right). Values are expressed as means ± standard deviation (*n* = 8). *—statistical significance at the level of *p* < 0.05 compared to SHAM; #—statistical significance at the level of *p* < 0.05 compared to CTRL; §—statistical significance at the level of *p* < 0.05 compared to ME50 group; £—statistical significance at the level of *p* < 0.05 compared to ME100 group.

The most pronounced lowering effect of superoxide anion radical (O_2_^−^) in the stabilization point was observed in the ME200 group compared to all other groups studied (*p* < 0.05). These baseline values were markedly lower in treated animals (ME50, ME100, and ME200) relative to CTRL and SHAM. However, in the first point of reperfusion, only ME50 significantly lowered O_2_^−^ in comparison with CTRL, but at the end of reperfusion in RP1 point, all three applied doses of ME significantly lowered the level of O_2_^−^ compared to CTRL, and these values were similar to SHAM (*p* < 0.05). At this point, ME200 seems to induce the most prominent decrease compared with the low and medium doses of ME, but this difference was not significant (Figure 2A). Seven-day preconditioning with lemon balm extract at a dose of 200 mg/kg significantly lowered the level of hydrogen peroxide (H_2_O_2;_ *p* < 0.05) compared to the CTRL group and groups of rats treated with a low and medium dose of the extract (ME50 and ME100). These differences were observed in all three points of interest (stab, RP1, and RP30; Figure 2B). However, low and medium doses of ME did not succeed in lowering the H_2_O_2_ reperfusion period and were significantly higher than SHAM. Index of lipid peroxidation measured as the thiobarbituric acid reactive substances (TBARS) was significantly decreased in all three experimental groups (ME50, ME100, and ME200) compared to the untreated control group of rats that had undergone global ischemia and SHAM group (*p* < 0.05). These differences were noticed in all three points of interest, during the stabilization period, first and last 30th min of the reperfusion period, with ME200 and ME100 being more efficient than the low dose ME50 (*p* < 0.05, Figure 2C). The level of nitrites (NO_2_^−^) was considerably increased in ME100 and ME200 compared to both SHAM, CTRL, and ME50 groups in the stabilization point (*p* < 0.05), while only ME200 elevated the level of NO_2_^−^ in the first and last minute of reperfusion period compared to all other groups studied (*p* < 0.05, Figure 2D).

**Figure 3:**
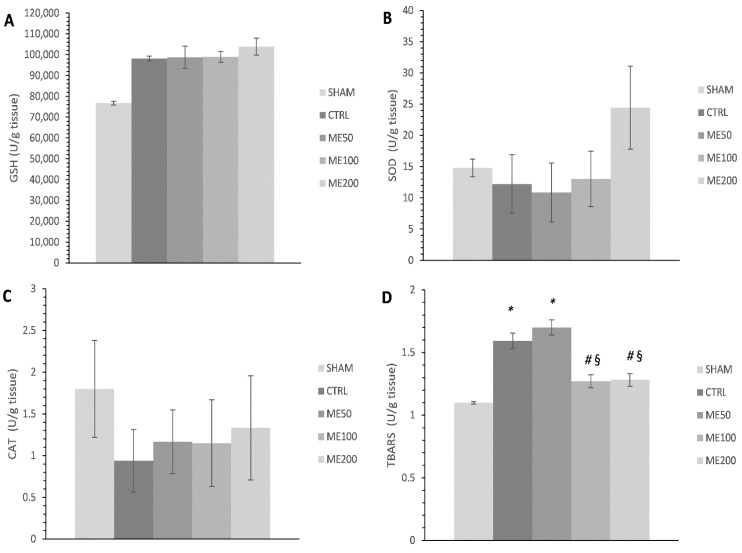
Effects of short-term heart preconditioning with ME on oxidative stress parameters in the heart tissue homogenate. (**A**) GSH—reduced glutathione; (**B**) SOD—superoxide dismutase; (**C**) CAT—catalase; (**D**) TBARS—index of lipid peroxidation measured as thiobarbituric acid reactive substances. Values are expressed as means ± standard deviation (*n* = 8). *—statistical significance at the level of *p* < 0.05 compared to SHAM; #—statistical significance at the level of *p* < 0.05 compared to CTRL; §—statistical significance at the level of *p* < 0.05 compared to ME50 group.

The application of the highest dose of ME has mildly increased the activity of superoxide dismutase SOD (Figure 3B) and the level of reduced gluthathione (GSH) in heart tissue homogenate when compared to the non-treated control group of rats (*p* > 0.05; Figure 3A), while no changes were observed in the activity of catalase (CAT) between studied groups (Figure 3C). Furthermore, the level of TBARS was significantly decreased in rats treated with high and medium doses of the extract (ME200 and ME100) when compared to both CTRL and ME50 groups of rats and was similar to the SHAM group of healthy rats (*p* < 0.05; Figure 3D). Additionally, CTRL and ME50 had significantly higher TBARS in relation to SHAM (*p* < 0.05).

### 2.4. Effects of Heart Preconditioning with ME on Heart Morphology

**Figure 4:**
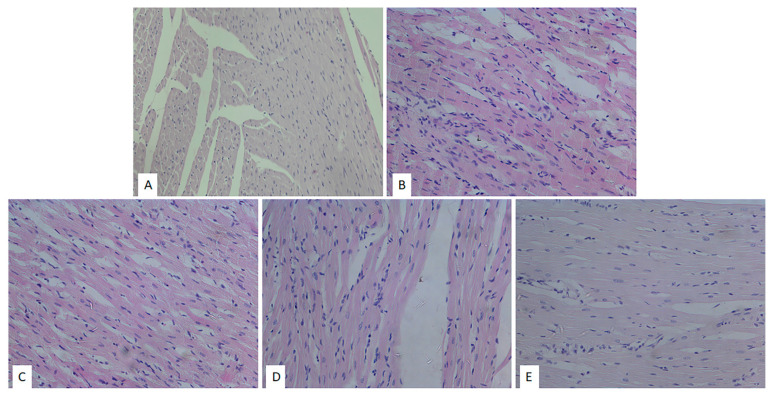
Effects of short-term heart preconditioning with ME on heart tissue morphology. Representative heart tissue sections of H/-E staining. Magnification 20× scale bar = 50 µm. (**A**) SHAM; (**B**) CTRL; (**C**) ME50; (**D**) ME100; (**E**) ME200.

The images of the heart tissues in the control group of rats were characterized by the presence of hypertrophy of individual muscle fibers with degenerative changes, stromae hypercellularity (cellular infiltrate), interstitial edema, and pycnotic nucleus. The appearance of wavy fibers was also observed (Table 1, Figure 4A). Similar changes (hypertrophy of individual muscle fibers, degenerative changes, hypercellularity of the stroma) were observed in the ME50 group of rats; however, these changes were slightly less noticeable in comparison to the non-treated CTRL group of rats (Table 1, Figure 4B). However, heart preconditioning with lemon balm extract at a medium dose of 100 mg/kg resulted in a reduction of degenerative alterations, hypercellular stroma, and interstitial edema compared to CTRL and ME50 groups (Table 1, Figure 1C). The degree of degenerative changes, as well as the presence of cellular infiltration and interstitial edema, was the least noticeable in the group of animals treated with ME at the highest dose of 200 mg/kg. Nonetheless, slightly altered nuclei were seen in the ME200 group (Table 1, Figure 1D).

**Figure 5:**
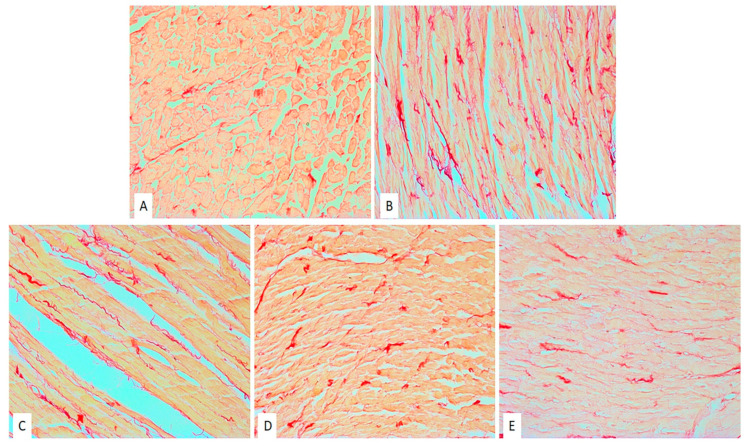
Effects of short-term heart preconditioning with ME on heart tissue collagen content. Representative heart tissue sections of Picrosirius red staining. Magnification 20× scale bar = 50 µm. (**A**) SHAM; (**B**) CTRL; (**C**) ME50; (**D**) ME100; (**E**) ME200.

**Figure 6:**
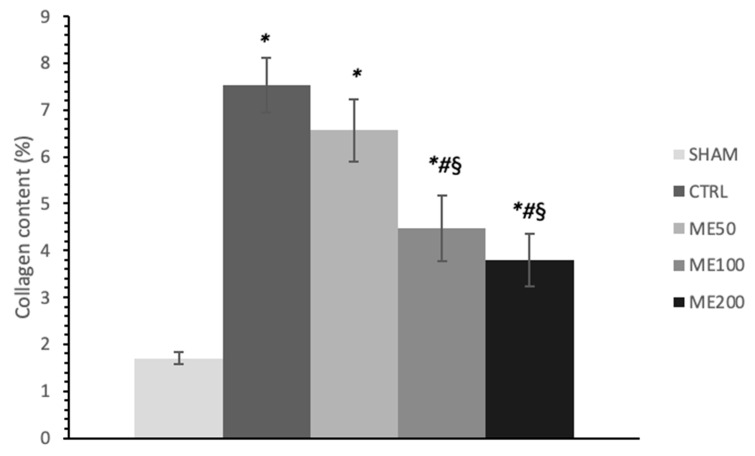
Effects of short-term heart preconditioning with ME on heart tissue collagen content expressed in percentage ±. Values are expressed as means ± standard deviation (*n* = 8). *—statistical significance at the level of *p* < 0.05 compared to SHAM; #—statistical significance at the level of *p* < 0.05 compared to CTRL; §—statistical significance at the level of *p* < 0.05 compared to ME50 group.

A significantly higher amount of fibrosis and collagen deposition was observed in the CTRL group of rat hearts subjected to global ischemia (Figure 5), while preconditioning with ME, in medium and especially in the highest dose (ME200) resulted in a significant almost two-fold reduction of collagen content relative to CTRL, ME50, and ME100 (*p* < 0.05). Additionally, treatment with the lowest dose of ME had no significant effect on collagen content compared to the CTRL. SHAM group of hearts had significantly lower collagen content than all other groups exposed to I/R injury (Figure 6; *p* < 0.05).

## 3. Discussion

Myocardial I/R injury stands for a phenomenon with a complex pathology involving many pathways inducing cardiomyocytes death via necrosis and apoptosis. Despite thousands of research pieces regarding the subject, the specific mechanisms of the I/R-induced cardiomyocyte damage have not been established yet. However, the available knowledge highlights the role of phytotherapeutics, including plant extracts and their active compounds, in ameliorating myocardial I/R injury in different ways [24]. This strategy refers to the prevention of irreversible cell death through preventing oxidative stress, calcium overload, and inflammation. Our study aimed to provide deeper insight into the cardioprotective properties of lemon balm extract and to investigate the role of oxidative stress as one of the key mechanisms of cell damage in this state. The specific goal was to investigate the possibility of short-term 7-day heart preconditioning with ME in three different doses to ameliorate myocardial I/R injury. Here, we showed that ME, especially at a dose of 200 mg/kg, was capable of improving cardiac function and reducing oxidative stress in the heart but also mildly boosting the antioxidant defense system in the myocardium.

The results of our study confirmed the well-known fact that exposition of the heart to global ischemia induces changes in cardiac function, in several aspects, including depression of contractile force, but also relaxation ability at the end of the reperfusion period, which is reflected by lower dp/dt max, dp/dt min, SLVP, and DLVP in the CTRL group of rats compared to non-ischemic SHAM rats. Additionally, ME treatment also affected the hearts` ability to stabilize, as proved via dp/dt max, dp/dt min, SLVP, HR, and CF improvement at the beginning of the protocol compared to CTRL rats. Moreover, the present study demonstrated the improvement of myocardial contractile function subjected to I/R injury after the treatment with lemon balm extracts, particularly in the ME200 group, via dp/dt max and dp/dt min increment. This observation is partly in line with the previous paper, where the administration of aqueous ME induced improvement of dp/dt max after isoproterenol-induced cardiac injury. However, they highlighted the best effect of ME in lower doses of 50 and 100 mg/kg, opposite to our findings where ME200 was found to be the most effective [20]. In our study, pre-treatment with ME also induced improvement of SLVP, especially in ME200. This finding does not entirely correlate with others. Namely, in the other study, ME50, ME100, and ME200 all experienced a mild decrease in SLVP. Even more, it was observed that lower doses, ME50 and ME100, decreased SLVP after isoproterenol-induced myocardial infarction compared to the same groups with no infarction [20]. The potential explanation for this divergence between our results and those published earlier may lie in the fact that different models were used to mimic myocardial infarction. The results regarding ME effects on the HR in the available literature are controversial. Namely, we found that all three doses of ME may decrease HR prior to ischemia in the stab point and, more importantly, that ME200 may decrease the HR at the beginning of reperfusion, with no effect on HR at the end of this period. Similar results were obtained in another study, where no changes in HR were noticed after 7-day treatment with aqueous ME in the same doses used in the present work (50, 100, and 200 mg/kg) [25]. On the contrary, other research provided evidence that HR decrement can be induced by a low dose of lemon balm extract (ME50) after the reperfusion period [26]. This can be explained by the fact that lemon balm may act negatively chronotropic in different ways: by blockage of K^+^ channels or by cardiac muscarinic receptors activation [27,28].

Sedighi et al. highlighted that a two-week application of lemon balm extract may ameliorate myocardial I/R injury induced by the LAD model. One of the proposed mechanisms is oxidative stress reduction via an increase in antioxidant enzyme SOD and a decrease in malondialdehyde (MDA), a product of lipid peroxidation, which was observed for the first time 5 days post-reperfusion. In our study, 7-day application of ME200 led to a mild but nonsignificant increase in SOD and GSH in the heart tissue homogenate, possibly due to the shorter time of exposition to ME and different kinds of samples used in the previously mentioned study compared to ours [26]. Nonetheless, we proved a significant decrease in the index of lipid peroxidation in the form of TBARS both in the heart tissue homogenate and in coronary venous effluent in all three points of interest, stabilization point, 1st, and 30th min of the reperfusion period, especially in medium and high dose of ME, which is in line with the previous results [26]. Additionally, we observed a significant drop in other ROS, such as O_2_^−^ and H_2_O_2,_ after preconditioning with ME. There is evidence that lemon balm extract may lower O_2_^−^ levels in different models of cardiac pathologies such as autoimmune myocarditis [28], and also in vitro can act cytoprotective against H_2_O_2_-induced oxidative stress in HUVECs by decreasing hydroperoxides concentration [29]. Interestingly, we found that ME induced an increase in the level of nitrites as an indirect indicator of nitric oxide (NO), correlating with elevated CF in treated animals, indicating the vasodilatory potential of ME. Others suggest that the aqueous ME may cause vasodilatation via a nitric oxide pathway in a model of isolated thoracic aorta, and the obtained results may be explained by this fact [30].

Cardioprotective effects that ME preconditioning displayed in this study are most probably the result of synergistic effects of its active components with rosmarinic acid as the leading component, which per se has been proved to exert an ameliorating effect on myocardial I/R injury in mice [14]. Additionally, flavonoid components of ME may have contributed to its beneficial effect on cardiac I/R injury, especially quercetin, which was the most abundant flavonoid component of our extract [23]. Quercetin was reported to improve cardiac contractility through dp/dt max improvement in isolated rat heart if acutely given either before, during ischemia, or during reperfusion period a decade ago [6], but it also improves oxidative stress parameters chronically in the 7-day treatment protocol [31].

Beneficial effects of ME were also observed in vitro on cardiomyocytes involving several mechanisms, including binding and activation of NF-kB, therefore inducing decreased ROS formation [13,32], prevention of LDH leakage, and preservation of ATP in the cells [33]. In vitro antioxidant effects of this extract were already explained in our previous research, including scavenging potential against DPPH radical, NO, OH, and FRAP, but also in vivo anti-inflammatory effect in carrageenan-induced paw edema model. These two properties may influence and be linked with the achieved cardiac effects in this study [23]. Alterations in myocardial architecture found in non-treated rats subjected to myocardial I/R injury were mostly prevented in the ME200 group, as shown in H/E-stained tissues, with no expanded interstitium, minor stromae hypercellularity and hypertrophy of cardiac cells, suggesting cytoprotective properties of the applied extract. However, insufficient evidence is available regarding the ME effects on cardiac morphology, except for the decrement in the infarct size after a 5-day reperfusion period, found in a study by Sedighi et al. [26]. Additionally, we found that 7-day preconditioning with ME200 achieved to prevent myocardial fibrosis induced by I/R injury, which is in line with our previous research in a different model of heart disease, autoimmune myocarditis [28]. This can partly be explained by the fact that rosmarinic acid exerts antifibrotic action in the heart via AMPKα/Smad3 signaling pathway, proved both in vivo and in vitro [34].

## 4. Materials and Methods

### 4.1. Ethical Concerns

All experiments and analyses conducted on laboratory animals or animal material used in this study were approved by the Ethics Committee for experimental animal well-being of the Faculty of Medical Sciences, University of Kragujevac (Kragujevac, Serbia) No.01-10171. Additionally, all the experimental procedures were performed following the European Directive2010/63/EU for the welfare of laboratory animals, number and principles of Good Laboratory Practice (GLP) (86/609/EEC). The experiments were carried out according to the European Union Directive 86/609/EES for the Protection of the Vertebrate Animals used for Experimental and other Scientific Purposes and the principles of ethics.

### 4.2. Preparation of the Melissa Officinalis Extract

The plant material used in this research, dried leaves of *Melissa officinalis* L. (*Lamiaceae*), was purchased from Bilje Borca, LLC, Belgrade, Serbia. The dried plant material was pulverized using a mill (IKA A11, IKA^®^ Werke GmbH& Co., Staufen im Breisgau, Germany), stored in well-sealed paper bags, and kept at room temperature until the extract was made. Ethanolic extract was made under the reflux of the solvent (70% ethanol) at the boiling point of the solvent (78.5 °C). The extraction process lasted 2.5 h. Afterward, the obtained mixture was filtered through gauze and left at room temperature to spontaneously precipitate ballast substances. Then, the obtained liquid extract was filtered (Whatman, No.1, Cytiva, Buckinghamshire, UK). Finally, a rotary vacuum evaporator (RV05 basic IKA, IKA^®^ Werke GmbH& Co., Staufen im Breisgau, Germany) at 40 °C, 90 rpm, and 250 mbar vacuum was used to obtain the dry extract. The dry extract was stored in dark glass vials at + 4 ºC until administration to animals. The extract was dissolved in tap water once daily prior to administration to animals per os [33].

### 4.3. Animals

The study involved a total of 40 eight-week-old male *Wistar albino* rats (body weight 300 ± 50 g) purchased from the Military Medical Academy Animal House, Belgrade. At first, animals were acclimatized for two weeks and kept in polyethylene cages (4 per cage) in quarantine under standardized, controlled environmental conditions (22 ± 2 °C and a 12-h light/dark cycle). During the whole period, animals had free access to standard food (9% fat, 20% protein, 53% starch) and water (ad libitum). Animals were randomly divided into the following groups:

SHAM—healthy rats given an equivalent amount of distilled water with no myocardial I/R injury (*n* = 8).

CTRL—healthy rats given an equivalent amount of distilled water with myocardial I/R injury (*n* = 8).

ME50—healthy rats treated with ME in a dose of 50 mg/kg with myocardial I/R injury.

ME100—healthy rats treated with ME in a dose of 100 mg/kg with myocardial I/R injury (*n* = 8).

ME200—healthy rats treated with ME in a dose of 200 mg/kg with myocardial I/R injury (*n* = 8).

ME was administered per os, once daily (every day at the same time) for 7 days. Prior to administration, the extract was dissolved in distilled water, and aqueous solution of ME was applied via gavage to rats. The volume was calculated according to the body weight of rats each day (approximately 300 μL).

### 4.4. Isolation of the Rat Heart and Langendorff Protocol

After 7 days of treatment with ME, on day 8 of the experimental protocol, animals were sacrificed. Firstly, animals were anesthetized by intraperitoneal injection of ketamine (10 mg/kg) and xylazine (5 mg/kg) and premedicated with heparin as an anticoagulant and sacrificed by decapitation. Afterward, the chest was opened via midline thoracotomy, the hearts were immediately removed and immersed in cold saline, and the aortas were mounted on the cannula of the apparatus and retrogradely perfused under a constant coronary perfusion pressure (CPP) of 70 cmH_2_O according to the Langendorff technique. Hearts were perfused with Krebs–Henseleit buffer with similar content as extracellular content (NaCl 118 mmol/L, KCl 4.7 mmol/L, CaCl_2_ × 2H_2_O 2.5 mmol/L, MgSO_4_ × 7H_2_O 1.7 mmol/L, NaHCO_3_ 25 mmol/L, KH_2_PO_4_ 1.2 mmol/L, glucose 11 mmol/L, pyruvate 2 mmol/L), equilibrated with 95% O_2_ plus 5% CO_2_, and warmed to 37 °C (pH 7.4).

### 4.5. Cardiodynamic Measurements

The sensor was placed (transducer BS473-0184, Experimetria Ltd., Budapest, Hungary) in the left ventricle, and the following parameters of myocardial function were measured: dp/dt max, dp/dt min, SLVP, DLVP, and HR. CF was measured flowmetrically. After stabilization (20–30 min) of the heart rhythm, the global ischemia was induced by stopping the perfusion through the heart, followed by 30 min reperfusion. All cardiodynamic parameters and coronary flow were measured in intervals of 5 min during the period of reperfusion (30 min) (RP1-RP7). In all of the mentioned points of interest, coronary venous effluent was collected for further analysis of oxidative stress.

### 4.6. Cardiac Redox State

Hearts from all animals were isolated, measured, and then frozen at −80 °C. Hearts were homogenized using an electrical homogenizer in PBS on ice (1:10, at pH 7.4). Then, the samples were centrifuged at 1200× *g* for 20 min at 4 °C, and the obtained supernatant was used for the spectrophotometric determination of the following oxidative stress parameters: index of lipid peroxidation (TBARS), NO_2_^−^ concentration, CAT, SOD activity and reduced GSH level (UV-1800, Shimadzu, Kyoto, Japan). Additionally, the following oxidative stress parameters (O_2_^−^, H_2_O_2_, NO_2_^−^, TBARS) were determined from the samples of venous coronary effluent according to our previous research [34].

### 4.7. Histological Analyses of the Heart (H/E Staining and Picrosirius Red Staining)

Immediately after the Langendorff protocol had finished, rat hearts were collected, measured, and cut into two halves so that both halves of the heart were available for further histological analysis. The hearts were fixed in 4% neutral paraformaldehyde; then, the tissues were dehydrated in increasing alcohol concentrations (70%, 96%, and 100%), cleared in xylene, immersed in paraffin, and prepared for subsequent analysis. Tissue sections (5 µm thick) were stained by the H/-E (Hematoxylin/Eosin) method in order to examine and confirm morphological changes and by the Picrosirius red staining in order to determine collagen content in the heart tissues. An Olympus BX51 light microscope (Bartlett, TN, USA) was used to capture the images of heart tissue sections.

Histological analysis of H/-E-stained tissue sections was based on the following criteria: presence (+) or absence (–) of degenerative changes, dilated interstitium, and hypercellularity. The intensity of changes was evaluated by using the following score: (–) no morphological changes, (+) mild (<10% per cross-section), (++) moderate (<20% per cross-section), and (+++) severe (<30% per cross-section) [35].

### 4.8. Statistical Analyses

Statistical analyses of the obtained data were performed using IBM SPSS 20.0 (IBM Corp., Armonk, NY, USA) for Windows. Kolmogorov–Smirnov, Shapiro–Wilk tests, histogram, and normal QQ plot tests were used to examine the normality of the distribution, while data were expressed as mean values (X) ± standard deviation (SD). The data were analyzed by one-way analysis of variance (ANOVA), followed by a post hoc Bonferroni test. This test is considered to be suitable for the determination of the statistical significance of three or more independent groups. Before applying the post hoc test, we tested the homogeneity of the variances between the groups (Levene’s test). If variances were homogeneous (*p* ≥ 0.05), the following multiple comparison method was selected Bonferroni [36]. A value of *p* < 0.05 was considered significant, while *p* < 0.01 was considered highly significant.

## 5. Conclusions

Hereby, we confirmed the capability of ME, especially in a high dose of 200 mg/kg, to significantly improve heart function and salvage the heart from I/R induced damage, even after short-term preconditioning. This refers to the prevention of cardiodynamic alterations, especially the improvement of contractility (via dp/dt max and dp/dt min), but also cardiac architecture in terms of ameliorating tissue changes and collagen deposition and fibrosis caused by I/R of the heart. Assumed mechanism of the achieved cardioprotective effects certainly implies oxidative stress amelioration, as proved by significant decrement of ROS production (O_2_^−^ and H_2_O_2_) and index of lipid peroxidation (TBARS). However, more mechanistic research is necessary to determine the precise signaling pathways involved. Achieved cardioprotection is assumed to be a consequence of the synergistic antioxidant and anti-inflammatory effect of ME constituents, mainly phenolic acid, rosmarinic acid, and flavonoid quercetin.

## Figures and Tables

**Table 1:** Histopathological changes of myocardial tissue depending on the degree, presence, or absence of degenerative changes, expanded interstitium, stromae hypercellularity, and muscle fibers hypertrophy.

Group	Degenerative Changes	Expanded Interstitium	Stromae Hypercellularity	Muscle Fibers Hypertrophy
*SHAM*	—	—	—	—
*CTRL*	*+++*	*+*	*+++*	*+++*
*ME50*	*++*	*+*	*++*	*++*
*ME100*	*++*	*-*	*++*	*++*
*ME200*	*+*	*-*	*+*	*+*

## Data Availability

Data is contained within the article and Supplementary Material.

## Supplementary Materials

The following supporting information can be downloaded at: https://www.mdpi.com/article/10.3390/ph15070840/s1, Figure S1: Chromatogram of *M. officinalis* ethanolic extract (ME); Table S1: Chemical composition of the investigated *M. officinalis* ethanolic extract (ME); Table S2: Validation parameters; Table S3: Phenolic and flavonoid content of the investigated *M. officinalis* ethanolic extract.
