# Supplementary material for: Short-Term Administration of Lemon Balm Extract Ameliorates Myocardial Ischemia/Reperfusion Injury: Focus on Oxidative Stress
Source: Pharmaceuticals (Basel). 2022 Jul 8;15(7):840. doi: 10.3390/ph15070840 (PMC9317599; doi:10.3390/ph15070840)
Supplement: Supplementary file 1 [file pharmaceuticals-15-00840-s001.zip › pharmaceuticals-1782293-supplementary.pdf]

SUPPLEMENTARY MATERIAL

Chemical profiling of the examined extract by means of high-performance liquid chromatography (HPLC)

Chemical characterization of the examined extracts and quantification of the selected compounds was performed by a validated HPLC method. Particularly, phenolic compounds were separated using an Agilent Technologies 1100 liquid chromatographer equipped with a diode array detector (*Agilent Technologies, Santa Clara, California, USA*). The components were separated using a reversed-phase Nucleosil C18 column (250 mm × 4.6 mm, 5 µm particle size; Agilent Technologies) held at 30 °C. Solvent A was 1% (v/v) aqueous HCOOH, and solvent B was methanol. The mobile phase was delivered in the gradient mode comprising 0 min 10% B, 10 min 25% B, 20 min 45% B, 35 min 70% B, 40 min 100% B, and 46 min 10% B. The HPLC mobile phase was prepared fresh daily and filtered through a nylon filter (pore size 0.45 µm). The injection volume was 10 µl, and the run time was 48 min using a variable flow rate (0–10 min, 1 ml·min-1; 10–20 min, 0.8 ml·min-1; 20–30 min, 0.7 ml·min-1; 30–46 min 1 ml·min-1) (Božin et al., 2013). For quantification of the selected compounds, chemical standards of gallic (GA), caffeic (CA), *trans*-cinnamic (CNA), *p*-coumaric (pQA), chlorogenic (CHA), rosmarinic (RA), and ferulic acid (FA), as well as quercetin (Qe), rutin (R), and quercitrin (Qt), were run under the same experimental conditions, using methanol as a solvent. The compounds of interest were monitored at 280 nm (GA, CA, and CNA), 330 nm (pQA, CHA, RA, FA, and Qe), and 350 nm (R and Qt). The experimental programs and data processes of quantification were both performed with the software Agilent OpenLAB Control Panel v.A.01.05 (*Agilent Technologies*). The results were expressed as milligrams per gram of dry extract.

Determination of phenolics and flavonoids content in the examined extract

Total phenolic content determination in the examined extracts was based on the spectrophotometric method, which involves the oxidation of polyphenolic compounds in the presence of Folin–Ciocalteu reagent (a mixture of phosphotungstic and phosphomolybdic acid) to phenoxide anions, and the reduction of reagents to tungsten oxide and blue molybdenum oxide. The intensity of the blue color is measured spectrophotometrically at $\lambda = 760$ nm and is directly proportional to the number of polyphenols in the examined samples of extracts. The total flavonoid content determination was based on the properties of flavonoids to build metal complexes with metals. In the reaction of flavonoids with $AlCl_3$, Al^{3+} binds to total flavonoids, and a complex of flavonoids with aluminum is formed. The intensity of the colored complex is determined spectrophotometrically at $\lambda = 415$ nm and is proportional to the flavonoid content in the tested sample.

Table S1. Chemical composition of the investigated *M. officinalis* ethanolic extract (ME)

**mg / g d.e.**																			
Phenolic acids														***Flavonoids***					
**trans-cinnamic acid**		**caffeic acid**		**p-coumaric acid**		**chlorogenic acid**		**rosmarinic acid**		**ferulic acid**		**gallic acid**		**Quercetin**		**rutin**		**quercitrin**	
**X**	**U**	**X**	**U**	**X**	**U**	**X**	**U**	**X**	**U**	**X**	**U**	**X**	**U**	**X**	**U**	**X**	**U**		**U**
1.34	0.15	0.45	0.02	0.34	0.03	8.78	0.44	109.44	6.57	0.36	0.02	0.11	0.02	1.08	0.08	<LOD		<LOD	

d. e.—dried extract; LOD—limit of detection; U—expanded measuring uncertainty with coverage factor k = 2.

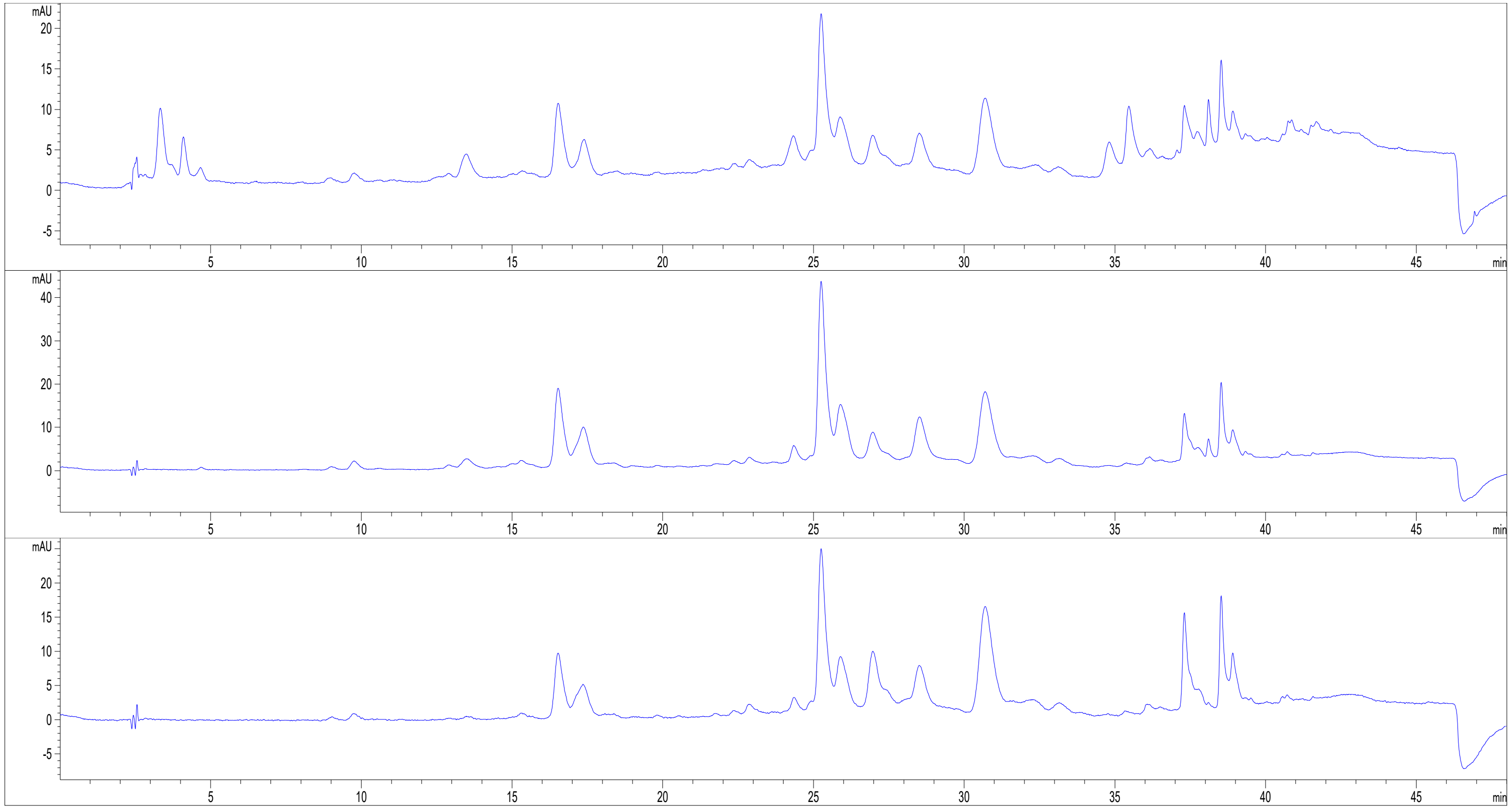

Figure S1. Chromatogram of *M. officinalis* ethanolic extract (ME) with detection at a) 280nm, b) 330nm, and c)350nm. Identified compounds: 1—trans cinnamic acid, 2—caffeic acid, 3—p-coumaric acid, 4—chlorogenic acid, 5—rosmarinic acid, 6—ferulic acid, 7—gallic acid, and 8—quercetin.

Table S2. Validation parameters.

Compound	Calibration curve	R2	Range (µg/mL)	LOD (µg/mL)	LOQ (µg/mL)	U* (%)
Gallic acid	y=3,0497x+5,4494	0.9986	0.3-18	0.06	0.3	15
p-Coumaric acid	y=5.5894x+12.473	0.9900	0.2-12	0.03	0.2	10
Caffeic acid	y=6.4807x+1.490	0.9999	0.2-12	0.03	0.2	7
Chlorogenic acid	y=2.9003x+4.687	0.9995	0.3-30	0.10	0.3	5
Rosmarinic acid	y=3.5125x+0.332	0.9983	0.5-30	0.15	0.5	6
trans-cinnamic acid	y=7.9398x+6.565	0.9997	0.1-6	0.05	0.1	11
Ferulic acid	y=1.6072x-5.512	0.9979	0.2-36	0.1	0.2	6
Quercitrin	y=2.4017x-2.8372	0.9995	0.5-30	0.15	0.5	5
Rutin	y=2.1224x+5.479	0.9959	0.5-30	0.20	0.5	8
Quercetin	y=9.4489x+8.399	0.9981	0.18-10.80	0.03	0.18	5

*Expanded measuring uncertainty with covering factor k=2

Table S3. *Phenolic and flavonoid content of the* investigated *M. officinalis* ethanolic extract.

Total phenolic content (mg GAE/g d.e.)	***Total flavonoid content (mg QE/g d.e.)***
X±SD	*X±SD*
73.19. ± 4.09	6.38± 0.49

GAE—gallic acid equvalents; d. e.—dried extract; QE—quercetin equivalents.

* This study is part of a larger project regarding therapeutic properties of *M. officinalis*; thus, the data such as provided chemical characterization (HPLC analysis, phenolic, and flavonoid content) of the extract have already been recently published in the Iranian Journal of Pharmaceutical Research (Reference in the manuscript file: *Draginic, N., Andjic, M., Jeremic, J., Zivkovic, V., Kocovic, A., Tomovic, M., Bozin, B., Kladar, N., Bolevich, S., Jakovljevic, V., Milosavljevic, I. et al. Anti-inflammatory and Antioxidant Effects of Melissa officinalis Extracts: A Comparative Study. Iran. J. Pharm. Res.* ***2022****, 21, e126561. doi: 10.5812/ijpr-126561).*
